# Differences in health behavior and nutrient intake status between diabetes-aware and unaware Korean adults based on the Korea national health and nutrition examination survey 2016–18 data: A cross-sectional study

**DOI:** 10.3389/fpubh.2022.1005369

**Published:** 2022-11-03

**Authors:** Anshul Sharma, Chen Lulu, Kee-Ho Song, Hae-Jeung Lee

**Affiliations:** ^1^Department of Food and Nutrition, College of Bionanotechnology, Gachon University, Seongnam-si, South Korea; ^2^Department of Internal Medicine, Konkuk University School of Medicine, Seoul, South Korea; ^3^Institute for Aging and Clinical Nutrition Research, Gachon University, Seongnam-si, South Korea; ^4^Department of Health Sciences and Technology, GAIHST, Gachon University, Seongnam-si, South Korea

**Keywords:** diabetes, nutrition education, health care, treatment, awareness

## Abstract

**Background:**

The aim of this study was to investigate the nutritional intakes and treatment regimens of Korean patients with type 2 diabetes who were aware of their condition.

**Methods:**

Participants (*n* = 16582) aged ≥ 19 years from the 2016–18 National Health and Nutrition Survey were divided into diabetes-aware and unaware groups and the variables were compared.

**Results:**

Among 1,906 (11.5%) diabetic adults, 1,433 (75.2%) were aware of their condition; 130 (9.1%) had nutrition education, and 1,340 (93.5%) were in the diabetes-aware treatment group. The diabetes-aware group had higher average age (*P* < 0.0001) and lower average BMI (*P* = 0.0015) than the unaware group. Intake of total fat (*P* = 0.0034), saturated fatty acids (*P* = 0.0021), riboflavin (*P* = 0.0035) and niacin (*P* = 0.0228) was significantly higher in the unaware group than in the diabetes-aware group, after adjusting energy intake for age and sex. Current smoking (*P* = 0.0046) and heavy drinking (*P* < 0.0001) rates were higher in the unaware group, whereas fiber intake (*P* = 0.0054) was lower in the unaware group. Higher levels of glycated hemoglobin were found in the group treated for diabetes (7.2%) than in the no-treatment (6.8%) group (*P* = 0.0048). Diabetes control was significantly better in the high income group.

**Conclusions:**

There is a need to strengthen nutritional education to prevent diabetes and improve the health status of diabetic patients in Korea.

## Introduction

Diabetes is a well-known chronic disease that can severely hamper quality of life. According to the International Diabetes Federation (IDF), the prevalence of diabetes among adults aged 20–79 years worldwide is estimated to increase to 9.6% in 2045 from 8.3% in 2019 (1). The prevalence of type 2 diabetes rises with age, with children under 18 years of age accounting for < 0.2% compared with over 25% among those over 65 years of age (2). According to the World Health Organization (WHO), approximately 1.6 million diabetes-related deaths occurred in 2016 worldwide, making diabetes the fourth leading cause of death due to non-infectious diseases (3). Similarly, in 2016, diabetes mellitus (DM) was the sixth prime cause of mortality in South Korea (4), with the disease afflicting approximately five million (14.4%) Koreans aged 30 years or older (5). As per nationally representative sample data of Korean individuals aged 30 years or older, the incidence rates of diabetes rose consistently over a 17-year period (from 2001 to 2018), from 8.6% to 13.8% (5–7). Additionally, the predicted number of Korean diabetics aged 30 years to 79 years rose from 3.20 million to 4.94 million in a period between 2010 and 2018 (8, 9). The prevalence of DM in Korean men and women has risen with age, with males in their 40s and females in their 50s witnessing a 10% surge (10). Also, the prevalence of prediabetes (fasting blood glucose (FBG) 100–125 mg/dL and/or glycated hemoglobin (hemoglobin A1c, HbA1c) at least 5.7% (39 mmol/mol) and 6.4% (48 mmol/mol)) has increased to 31.0 and 19.7% in Korean men and women, respectively, aged 30 years and over (11). Westernized lifestyles and lack of awareness likely contribute to this increased disease burden, in part due to the abundance of processed foods and sedentary routine facilitated by technology. Current evidence suggests that prolonged sedentary lifestyle is a risk factor for diabetes and cardiovascular ailments (12). Further, the healthcare and socioeconomic burden in society is increased by diabetes-related macro- and microvascular comorbidities such as retinopathy, nephropathy, and cerebrovascular disease (13).

Many individuals affected by diabetes are not aware of their diabetic status, and therefore do not visit medical centers promptly to access adequate health care (14). Medical costs, social support access and self-efficacy, are some of the factors that affect treatment adherence (15). In Korea, only 8.4% of individuals with diabetes have a reasonable control of their HbA1c, lipids, and blood pressure levels. These metrics measured by the Korea Diabetes Association (KDA) reflect poor health behavior and decisions (5). Further, approximately 60% of Koreans never received education in diabetes care (16). According to the Korea National Health and Nutrition Review Survey (KNHANES) results, the level of diabetes awareness among individuals with diabetes decreased from 70.7% in 2013–14 to 62.6% in 2013–16 (5, 17). Further, Korean women aged ≥ 30 years are more aware (65.8%) of their diabetes status than men (59.9%) of the same age group (5). Another study based on 2007–09, 2010–12, 2013–15, and 2016–17 KNHANES data concluded that the crude diabetes awareness rate was 72.3% and the levels of impaired FBG increased by 10% approximately (18).

Diabetes education in Korea is currently implemented in health centers by an outreach team composed of physicians, nurses, and clinical nutritionists (19). In addition, the Diabetes Quality Assessment program is currently being introduced in primary care hospitals, but not in general hospitals, where about 30% of the Korean population is monitored (20). Poor nutrition contributes to several chronic ailments, including diabetes. However, while medications are recommended by doctors for continued diabetes care, nutrition education is generally ignored (21). Nutrient intake findings among Korean diabetic patients suggest that the proportion of daily consumption of carbohydrates is ≥ 60% higher in women than in men (22, 23). A diet that is high in processed foods, manufactured with added sugars and other refined carbohydrates, is a principle factor driving the growing epidemics of DM (24). Conversely, a diet rich in whole grains and whole grain foods, including breakfast cereal, whole grain oats, brown rice, etc., was significantly associated with a lower risk of DM (25, 26). Regular intake of excess carbohydrates raised HbA1c in Korean women (27). According to the KNHANES 2008–13 study, the proportion of diabetes subjects who had received nutrition education was 14.4% in the well-controlled group and 10.2% in the uncontrolled group (19). According to a recent study (KNHANES 2012–13), the calorie intake of the unaware group in the Korean Reference Nutrient Intake (RNI) was significantly higher than in the aware group (28).

These findings underscore the need for additional studies based on national representative data to explore the awareness of diabetes, treatment and nutrition education in diabetes-aware population in Korea. Thus, to update the information correlating diabetes awareness and nutrient intakes among Korean diabetic adults, reported in 2012–13 (28), this cross-sectional study analyzed the differences in health behaviors and nutrient intake status among aware and unaware Korean adults with diabetes using 2016–18 KNHANES data. The study focuses on the following objectives by comparing: (1) differences in health behavior and nutrient intake according to diabetes; (2) differences in health behavior and nutrient intake according to diabetes awareness among diabetic patients; (3) the difference in health behavior and nutrient intake according to nutrition education or treatment among people who were aware of diabetes. We assessed general characteristics by examining information on age, gender, body mass index (BMI), smoking and drinking levels, nutritional intake, and nutrition education between diabetes-aware and non-aware groups based on released data from KNHANES. We further assessed the characteristics based on diabetes awareness among Korean subjects and nutrition education and treatment among diabetes-aware group. This national level data analysis provides useful insights into diabetes management in Korea.

## Research design and methods

### Data source and study design

**Figure 1:**
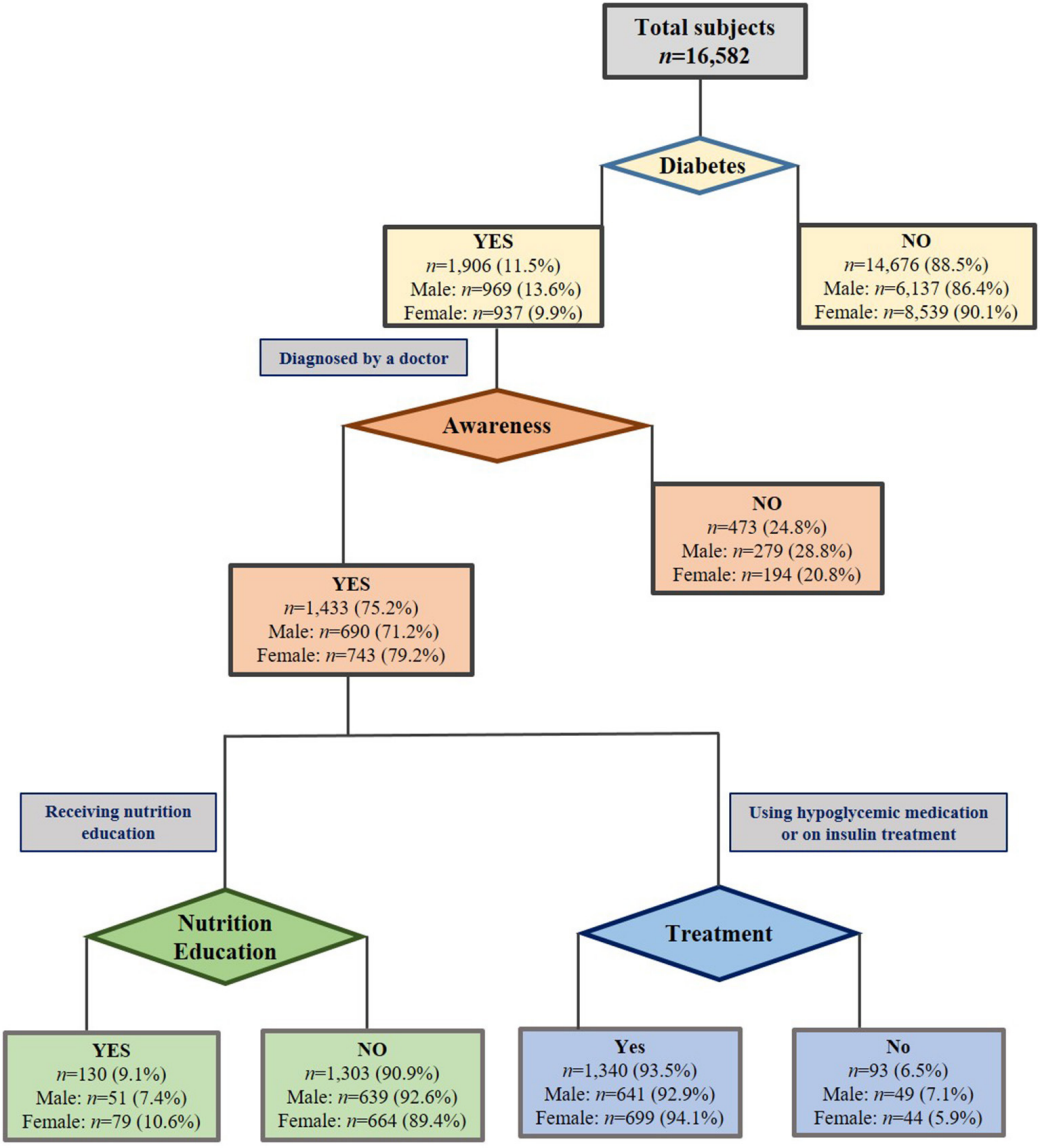
Distribution of individuals with diabetes in Korean adults.

The cross-sectional study was based on the 2016–18 KNHANES data. We excluded pregnant and lactating women and subjects whose energy intake per day was < 500 kcal and > 6,000 kcal, respectively. The legal adult in Korea is 19 years of age or older. So, our analysis included 16,582 participants aged 19 years and over (Figure 1), including 42.9% males. They were categorized into four main groups: (1) diabetes (1,906 subjects including 969 men and 937 women), and non-diabetes group (14,676 subjects including 6,137 men and 8,539 women) diagnosed by a physician; (2) the aware group (1,433 subjects including 690 men and 743 women), and the unaware group (473 subjects including 279 men and 194 women), identified as persons who, at the time of the measurement, were not clinically diagnosed but had eight-hour FBG > 126 mg/dL or HbA1c > 6.5% (49 mmol/mol), (3) those among diabetes aware group who received nutrition education or counseling (130 including 51 men and 79 women), not received nutrition education (1,303; 639 men and 664 women), and (4) subjects in the diabetes-aware group who underwent treatment (1,340 including 641 men and 699 women) for hypoglycemia whether or not using insulin (93 including 49 men and 44 women), defined by a physician. Since the data used in our study has been archived in a public depository, it is also publicly available and ethical clearance is not needed. The IRB number for the released data from the KNHANES is 2018-01-03-P-A.

### Variables of health, health interview, and examination

The KNHANES information is compiled based on a health interview, a health inspection, and a nutrition survey. Clinical assessment and health evaluation were performed in a mobile testing center by qualified medical personnel and interviewers (29). Demographics including age, sex, BMI, muscle strength practice, drinking and smoking patterns, income level, education level, muscular strength exercise, and regular aerobic exercise, were gathered from the KNHANES. Education levels were categorized into four types: elementary, middle, high school, and college or higher levels. The muscle strength practice was classified into three levels: not at all, 1–3 days a week, and ≥ 4 days/week. Drinking pattern was classified into four categories: non-drinkers, less than once a month, once a month less than heavy drinker, and heavy drinker. The income level was ranked into low, middle-low, middle-high, and high. Other parameters included: age, sex (male/female), and regular aerobic exercise (yes/no). Based on the definition of variables used in KNHANES survey, the proportion of subjects practicing strength exercises such as weights, iron bars, dumbbells, push-ups, and sit-ups for more than 2 days in the last week were determined. A medium-intensity physical exercise was performed for 2 h and 30 min or more. High-intensity physical activity lasted for 1 h and 15 min, or a combination of 2 min of medium-intensity, 1 min of high intensity physical activity per week.

### Nutrient assessment

Trained dietitians collected the nutrition survey data at their home. We used nutrient intake data of 1–day 24–h recall released from KNHANES.

### Statistical analysis

Data representing reference population from the 2016–18 KNHANES were analyzed using SAS software version 9.4 (SAS Institute Inc., Cary, NC, USA). Statistical analysis was conducted after adjusting for the KNHANES sample weights, strata and clusters and estimated standard errors (SE) reflecting survey design (SAS syntax as “proc SURVEYMEANS, proc SURVEYFREQ and proc SURVEYREG”). Health behaviors and socioeconomic status between groups were adjusted for age and sex. The nutrient intake was adjusted additionally for energy. The statistical significance was tested at *P* < 0.05.

## Results

### General characteristics

Table 1 presents the general characteristics of participants with and without diabetes, diabetes-aware and unaware groups, and the nutrition education and treatment among awareness. The average age and BMI in the diabetes group were 60.9 ± 0.4 years and 25.6 ± 0.1 kg/m^2^, respectively, compared with 46.0 ± 0.2 years and 23.8 ± 0.4 kg/m^2^, respectively, in the non-diabetic group (*P* < 0.0001). Compared with the control group, the diabetic group comprised a higher percentage of male subjects (*P* < 0.0001), while the rates of aerobic (*P* < 0.0001) and muscle exercise (*P* = 0.0001) were lower in diabetic individuals. In the diabetes group, the ratio of non-drinkers was high, but the proportion of heavy drinkers was higher, and the current smoking rate was low. Lower education was correlated with lower income in individuals affected by diabetes. Compared with the unaware group, the aware group consisted of subjects with a higher mean age (*P* < 0.0001), a lower proportion of male individuals (*P* < 0.0001) and lower BMI (*P* = 0.0015). The ratios of current-smokers (*P* = 0.0046) and heavy drinkers (*P* < 0.0001) were higher in the diabetes-aware group than in the unaware group. Among 1,433 individuals with diabetes, 130 received nutrition education (9.1%). No significant difference was observed between the two groups (Table 1). In the aware group, the proportion of treated individuals was 93.5% compared with untreated group (6.5%). Compared with the treated group, the untreated group of subjects tended to show a lower mean age, a higher proportion of men, a lower average income, a higher rate of smoking and drinking, and a higher proportion of individuals who were practicing muscle exercise and aerobic physical activity. No significant difference was observed between the two groups (Table 1).

**Table 1:** Demographic characteristics by diabetes, awareness of diabetes, nutrition education and treatment or not in Korean adults.

		**Diabetes**	**Non-diabetes**	***P*- value**	**Aware group**	**Unaware group**	***P*-value**	**Diabetes aware group**					
								**Nutrition education**	**No education**	***P*-value**	**Treatment**	**No treatment**	***P*-value**
		***n* = 1,906**	***n* = 14,676**		***n* =1,433**	***n* = 473**		***n* = 130**	***n* = 1,303**		***n* = 1,340**	***n* = 93**	
Age	MEAN±SE	60.9 ± 0.4	46.0 ± 0.2	< 0.0001	63.9 ± 0.4	53.4 ± 0.8	< 0.0001	64.9 ± 1.4	63.8 ± 0.4	0.4586	64.2 ± 0.4	59.8 ± 1.8	0.0143
Sex	Male	969 (50.8)	6137 (41.8)	< 0.0001	690 (48.1)	279 (59.0)	< 0.0001	51 (39.2)	639 (49.0)	0.1125	641 (47.8)	49(52.7)	0.6455
	Female	937 (49.2)	8539 (58.2)		743 (51.9)	194 (41.0)		79 (60.8)	664 (51.0)		699 (52.2)	44 (47.3)	
BMI	MEAN±SE	25.6 ± 0.1	23.8 ± 0.4	< 0.0001	25.2 ± 0.1	26.7 ± 0.2	0.0015	25.6 ± 0.3	25.1 ± 0.1	0.7013	25.1 ± 0.1	25.2 ± 0.3	0.8201
Smoking	Current-smoker	324 (17.0)	3113 (21.2)	< 0.0001	220 (15.4)	104 (22.0)	0.0046	18 (13.9)	202 (15.5)	0.6678	202 (15.1)	18 (19.4)	0.1523
	Past-smoker	540 (28.3)	2826 (19.3)		407 (28.4)	133 (28.1)		19 (22.3)	378 (29.0)		379 (28.3)	28 (30.1)	
	Non-smoker	1042 (54.7)	8737 (59.5)		806 (56.3)	236 (49.9)		83 (68.4)	723 (55.5)		759 (56.6)	47 (50.5)	
Alcohol drinking	Non-drinking	356 (18.7)	1484 (10.1)	< 0.0001	298 (20.8)	58 (12.3)	< 0.0001	36 (27.7)	262 (20.1)	0.1026	281 (21.0)	17 (18.3)	0.5729
	Less than once a month	171 (9.0)	2018 (13.8)		114 (8.0)	57 (12.1)		7 (5.4)	107 (8.2)		105 (7.8)	9 (9.7)	
	Once a month–less than heavy drinker	1169 (61.3)	9746 (66.4)		885 (61.8)	284 (60.0)		80 (61.5)	805 (61.8)		831 (62.0)	54 (58.1)	
	Heavy drinker	210 (11.0)	1428 (9.7)		136 (9.5)	74 (15.6)		7 (5.4)	129 (9.9)		123 (9.2)	13 (13.9)	
Muscular strength exercise	None	1591 (83.5)	10720 (77.4)	0.0001	1027 (84.2)	384 (81.2)	0.2693	105 (80.8)	1102 (84.6)	0.4306	1133 (84.6)	74 (79.6)	0.4241
	1-3 Day/Week	136 (7.1)	1847 (13.3)		92 (6.4)	44 (9.3)		12 (9.2)	80 (6.1)		85 (6.3)	7 (7.5)	
	≥4 Day/Week	179 (9.4)	1286 (9.3)		134 (9.4)	45 (9.5)		13 (10.0)	121 (9.3)		122 (9.1)	12 (12.9)	
Regular aerobic exercise	No	1223 (67.2)	7396 (55.9)	< 0.0001	941 (68.4)	282 (63.2)	0.4205	80 (63.6)	861 (68.9)	0.8632	885 (68.8)	56 (62.9)	0.0666
	Yes	598 (32.8)	5828 (44.1)		434 (31.6)	164 (36.8)		46 (36.5)	388 (31.1)		401 (31.2)	33 (37.1)	
Income	Low	565 (29.8)	3581 (24.5)	0.0001	412 (28.8)	153 (32.6)	< 0.0001	39 (30.0)	373 (28.7)	0.3839	371 (27.8)	41 (44.1)	0.0284
	Middle-low	488 (25.7)	3617 (24.7)		365 (25.5)	123 (26.2)		30 (24.6)	333 (25.6)		349 (26.1)	16 (17.2)	
	Middle-high	445 (23.4)	3725 (25.5)		347 (24.3)	98 (20.9)		26 (20.0)	321 (24.7)		324 (24.3)	23 (24.7)	
	High	401 (21.1)	3715 (25.4)		305 (21.3)	96 (20.4)		33 (25.4)	272 (20.9)		292 (21.9)	13 (14.0)	
Education	Elementary school	724 (39.8)	2508 (18.9)	< 0.0001	609 (44.3)	115 (26.0)	< 0.0001	54 (43.6)	555 (44.4)	0.4698	573 (44.6)	151 (28.3)	0.2658
	Middle school	291 (16.0)	1238 (9.4)		230 (16.7)	61 (13.8)		19 (15.3)	211 (16.9)		218 (17.0)	73 (13.7)	
	High school	496 (27.3)	4239 (32.0)		353 (25.7)	143 (32.3)		36 (29.0)	317 (25.3)		325 (25.3)	171 (32.1)	
	College or higher	307 (16.9)	5254 (39.7)		183 (13.3)	124 (28.0)		15 (12.1)	168 (13.40)		169 (13.2)	138 (25.9)	

Adjusted for age and sex.

### Nutrient intake

A comparison of nutrient intake according to diabetes status, diabetes awareness and nutrition education and treatment, among diabetes-aware group is presented in Table 2. The intakes of total calories (*P* = 0.0105), cholesterol (*P* = 0.0002), and sugar (*P* = 0.0001) were lower in the diabetes group than in the non-diabetes group. Fiber consumption was higher in the diabetes group than in control without any significant difference. After adjusting for age and sex, intakes of total fat (*P* = 0.0034) and saturated fatty acids (*P* = 0.0021) were found to be higher in the unaware group than in the aware group. Compared with the aware group, dietary fiber intake was lower in the unaware group (*P* = 0.0054). Niacin and riboflavin intakes were significantly higher in the unaware group (*P* = 0.0228, *P* = 0.0035, respectively) than in the diabetes-aware group. Sodium intake was lower in the aware group than in the unaware control. There was no significant difference between groups with and without nutrition education among the diabetes-aware group.

**Table 2:** Nutrient intake according to diabetes, aware and unaware, based on nutrition education and treatment or not in Korean adults.

	**Diabetes**	**Non-diabetes**	***P-*value**	**Awareness**	**No Awareness**	***P*-value**	**Diabetes aware group**					
							**Nutrition education**	**No-education**	***P*-value**	**Treatment**	**No treatment**	***P*-value**
	***n* = 1,906**	***n* = 14,676**		***n* = 1,433**	***n* = 473**		***n* = 130**	***n* = 1,303**		***n* = 1,340**	***n* = 93**	
Energy (Kcal)	1889.9 ± 25.2	2036.3 ± 11.3	0.0105	1798.4 ± 26.4	2117.4 ± 53.7	0.1833	1623.3 ± 77	1814.9 ± 27.1	0.1089	1788.7 ± 26.4	1912.7 ± 108.6	0.651
Protein (g)	64.8 ± 1	73.7 ± 0.5	0.1003	60.7 ± 1.1	75 ± 2.1	0.1031	54.4 ± 3.2	61.3 ± 1.1	0.7859	898 ± 19	69 ± 5.3	0.1042
Total fat (g)	36.2 ± 1	47.1 ± 0.4	0.8237	31.7 ± 0.8	47.2 ± 2.5	0.0034	26.8 ± 2	32.2 ± 0.9	0.3404	31.4 ± 0.9	36.4 ± 3.7	0.6654
Fatty acid (g)	11.2 ± 0.3	15.3 ± 0.2	0.4000	9.6 ± 0.3	15 ± 0.8	0.0021	8.4 ± 0.7	9.8 ± 0.3	0.8246	9.5 ± 0.3	11.3 ± 1.4	0.6126
MUFA* (g)	11.3 ± 0.4	15.1 ± 0.2	0.7976	9.8 ± 0.3	15.1 ± 0.9	0.0101	7.9 ± 0.8	9.9 ± 0.3	0.2256	9.6 ± 0.3	11.1 ± 1.3	0.8771
PUFA† (g)	10.2 ± 0.2	12.1 ± 0.1	0.7140	9.2 ± 0.2	12.4 ± 0.6	0.0717	7.9 ± 0.4	9.4 ± 0.2	0.3091	9.2 ± 0.2	9.8 ± 1.1	0.7859
n-3 Fatty acid (g)	1.8 ± 0.1	1.9 ± 0	0.4970	1.7 ± 0.1	2 ± 0.1	0.2579	1.4 ± 0.1	1.7 ± 0.1	0.2364	1.7 ± 0.1	1.5 ± 0.1	0.0211
n-6 Fatty acid (g)	8.4 ± 0.2	10.2 ± 0.1	0.5488	7.6 ± 0.2	10.4 ± 0.5	0.0765	6.5 ± 0.4	7.7 ± 0.2	0.5224	7.5 ± 0.2	8.3 ± 1	0.8707
Cholesterol (mg)	181.6 ± 5.8	252.4 ± 2.6	0.0002	160 ± 6	235.2 ± 12.8	0.0529	129.6 ± 14.9	162.9 ± 6.4	0.4011	159.3 ± 6.3	167.7 ± 21.2	0.3988
Carbohydrate (g)	295.5 ± 3.4	299.8 ± 1.5	0.0512	291 ± 3.8	306.6 ± 7.1	0.073	279.3 ± 10.7	292.1 ± 4	0.1232	291.6 ± 3.9	284.2 ± 10.9	0.0842
Fiber (g)	26.8 ± 0.4	25 ± 0.2	0.6935	27 ± 0.5	26.2 ± 0.8	0.0054	25.7 ± 1.6	27.1 ± 0.5	0.7656	26.8 ± 0.5	29.4 ± 1.6	0.1781
Sugar (g)	53 ± 1.2	63.3 ± 0.6	0.0001	50.8 ± 1.1	58.7 ± 2.9	0.8291	50.5 ± 3.6	50.8 ± 1.2	0.3226	50.2 ± 1.1	57.8 ± 4.7	0.286
Calcium (mg)	496.4 ± 8.9	523.1 ± 3.9	0.1990	475.9 ± 9.8	547.4 ± 18	0.711	467.7 ± 27.4	476.7 ± 10.3	0.2059	473.5 ± 9.7	503.8 ± 46	0.9604
Phosphorus (mg)	1019 ± 14	1087.9 ± 5.9	0.2616	972.7 ± 14.6	1134 ± 28.9	0.3484	927.6 ± 44.1	977 ± 14.9	0.1436	968.2 ± 14.6	1026.6 ± 65.7	0.9426
Iron (mg)	12 ± 0.2	12.2 ± 0.1	0.4046	11.6 ± 0.2	12.9 ± 0.4	0.9104	11.2 ± 0.6	11.7 ± 0.2	0.2109	11.6 ± 0.2	11.5 ± 0.7	0.4275
Sodium (mg)	3355.2 ± 59.7	3491.6 ± 25	0.1624	3223.2 ± 66.9	3683.5 ± 121.6	0.1488	2798.9 ± 155.7	3263.3 ± 71.1	0.3693	3199.9 ± 69.4	3499.4 ± 231.3	0.8125
Potassium(mg)	2820.3 ± 40.1	2856 ± 16.7	0.1637	2769.1 ± 45.2	2947.5 ± 77.4	0.078	2575.5 ± 133.5	2787.4 ± 45.9	0.9763	2751.4 ± 43.5	2979 ± 188.2	0.4587
Vitamin A (μgRE)	609.5 ± 46.9	612.6 ± 6.8	0.7195	524.3 ± 13.7	821.2 ± 157.5	0.0736	481.4 ± 32	528.4 ± 14.5	0.9186	523.6 ± 13.8	532.5 ± 59.2	0.6775
Vitamin A (μgRAE)	360.2 ± 26.9	383.3 ± 4.7	0.825	302.8 ± 7.9	503 ± 89.9	0.0541	277.8 ± 18.1	305.1 ± 8.4	0.9516	303.2 ± 8.1	297.9 ± 32.7	0.3812
Carotene (μg)	2996 ± 262.8	2757 ± 32.4	0.6132	2662.4 ± 73.8	3825.2 ± 889.8	0.2988	2447.5 ± 184.8	2682.7 ± 78.5	0.8919	2648.5 ± 73.7	2827.3 ± 325	0.8757
Retinol (μg)	110.1 ± 14.5	153.1 ± 3.5	0.6584	80.6 ± 3.8	183.7 ± 48.5	0.0988	73.5 ± 10	81.3 ± 4.1	0.913	82.2 ± 4.1	61.2 ± 11.7	0.0137
Thiamine (mg)	1.3 ± 0	1.4 ± 0	0.5902	1.2 ± 0	1.5 ± 0.1	0.1234	1.1 ± 0	1.2 ± 0	0.3371	1.2 ± 0	1.4 ± 0.1	0.219
Riboflavin (mg)	1.4 ± 0	1.6 ± 0	0.1878	1.3 ± 0	1.7 ± 0.1	0.0035	1.2 ± 0.1	1.3 ± 0	0.8667	1.3 ± 0	1.4 ± 0.1	0.5279
Niacin (mg)	12.3 ± 0.2	13.9 ± 0.1	0.1299	11.4 ± 0.2	14.5 ± 0.5	0.0228	10.9 ± 0.7	11.5 ± 0.2	0.3616	11.4 ± 0.2	12 ± 0.8	0.7355
Vitamin C (mg)	60.6 ± 2.1	63.1 ± 1	0.4403	56.2 ± 1.8	71.3 ± 5.2	0.134	50 ± 5.1	56.8 ± 1.9	0.5469	55.5 ± 1.9	64.7 ± 5.8	0.3638

Energy intake was adjusted for age and sex and other variables were adjusted for age, sex, and energy intake except energy intake.

*MUFA, Monounsaturated fatty acid; †PUFA, polyunsaturated fatty acid.

Total energy and cholesterol intake appeared to be higher in diabetes-aware individuals without nutrition education. The intake of carbohydrates and sugar was less in the nutrition-educated group than in the uneducated group. Vitamins and mineral intake was lower in the educated group. The intake of dietary fiber was 25.7 g in individuals with nutrition education compared with 27.1 g for those without nutrition education (*P* = 0.7656).

Among patients with diabetes awareness, the untreated group had a higher intake of energy than the treated group. The intakes of carbohydrate, omega 3 fatty acids, and retinol were higher in the treated group than in untreated group (Table 2). After adjusting for age, sex, and energy, *P*-values for intakes of dietary fiber (*P* = 0.1781) and sugar (*P* = 0.286) were less and carbohydrate intake (*P* = 0.0842) was higher in the treated group than in the control group. Additional statistical analysis was performed after adjusting for common confounding variables: (1) age, sex, energy, and income (2) age, sex, energy, income, and exercise (3) age, sex, energy income, smoking, drinking, exercise, and education. There was no change in the level of significance after adjusting for confounders.

### HbA1c analysis in diabetes-aware group

**Figure 2:**
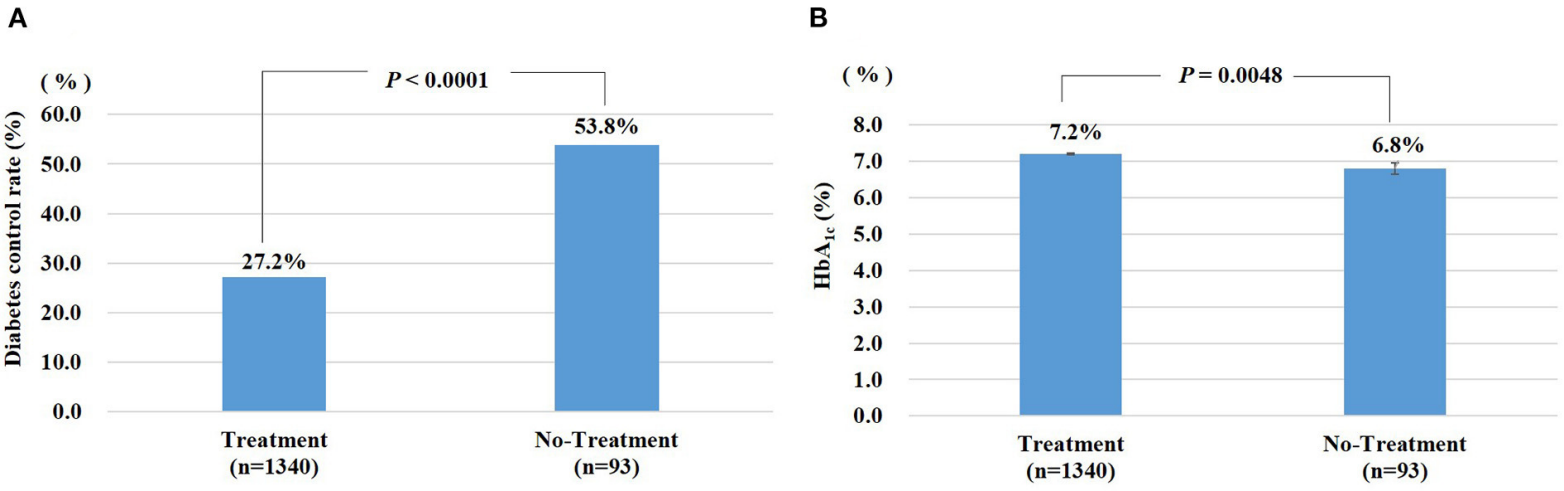
**(A)** Diabetes control rate (%): percentage of diabetic patients whose HbA_1c_ is less than 6.5%. **(B)** HbA_1c_ was expressed as means ± standard error (STDERR). Treatment: Diabetic patients are defined as taking hypoglycemic agents or insulin. No-Treatment: Diabetic patients who are not taking hypoglycemic agents or insulin.

Among the 1,433 diabetes-aware subjects, 93.5% were treated with drugs. Patients with diabetes whose disease was considered “controlled” were defined by HbA1c < 6.5% (39 mmol/mol). The proportion of individuals with controlled disease was 27.2% in the treated group and 53.8% in the untreated subjects who were aware of their diabetes status (*P* < 0.0001). Among subjects receiving drug treatment in the diabetes-aware group, the HbA1c ≥ value of those in the treated group was 7.2%, compared with 6.8% of the untreated group (*P* = 0.0048) (Figure 2).

## Discussion

In this study, we present differences in health behavior and nutrient intake status among diabetes-aware and unaware Koreans based on 2016–18 KNHANES data, a representative sample of adult Koreans. The study assessed the effect of nutrition education and treatment on diabetes-aware and unaware Koreans in enhancing knowledge and practice for the prevention and management of diabetes.

This study showed that individuals in the diabetes group tend to be older, have higher BMIs, and a higher likelihood to have a history of smoking. In a previous study, a data analysis of 1,476 subjects aged 45–74 who participated in the 2006–07 (Etude Nationale Nutrition Santé) study in France (*n* = 1,476 including 101 patients with diabetes) showed that those with diabetes tended to be older than healthy individuals, were most likely to have smoked, and likely had higher BMIs (30). As shown in Table 1, the diabetes group consisted of significantly higher proportion of lower-income individuals with elementary level education, which prevented access to nutrition education or healthier and more expensive foods. As a result, these individuals consumed whatever was easily accessible and affordable to them. A 2000–08 cross-sectional study involving 27,090 residents from Saskatchewan, Canada, reported that lower household income was found to be strongly associated with higher prevalence of diabetes (31). Income is a key yet understudied factor in type 2 diabetes, and suggests the need for further research and prompt policy intervention. In Korea, the increased incidence of diabetes was mainly attributed to aging inhabitants (32). This phenomenon could be immune-mediated, since smoking, aging, and poor nutrition lead to immune dysfunction and maladaptive immune responses, which increases the risk of diabetes. Therefore, in particular, given the incidence and diabetes-related diseases in the elderly, diabetes treatment is important to protect the vulnerable elderly population (5). Consistent with a recent study (28), a significant difference exists between age and BMI among diabetes-aware and unaware groups, with a higher age and lower BMI reported in the diabetes-aware group. According to a survey of primary care, 77% of patients with recently confirmed diabetes showed a BMI > 23.0 kg/m^2^ (33). An increase in BMI is a risk factor for prediabetic and diabetic conditions (11). Thus, weight control is necessary to avoid progression from prediabetes to diabetes and to regulate blood glucose levels in type 2 diabetes (27, 34). Overall, the mean intakes of energy, total fat, fatty acids, cholesterol, carbohydrates, and sugar are lower in the diabetes-aware groups than in unaware groups (28). Average fiber intake was greater in the diabetes-aware group than in the unaware group (*P* = 0.0054). However, the intake of dietary fiber was lower in diabetes-aware subjects exposed to nutrition education and treatment compared with controls without any significant difference. These discrepancies can be explained by the fewer subjects in the educated group and possible lower nutritional awareness in the treated group. In previous studies, dietary fiber intake reduced HbA1c by 0.55% in type 2 diabetes patients (35) and lowered the risk of diabetes in a healthy Japanese population (36). To this end, adopting a healthy dietary habit and increasing public awareness across all generations of diabetes are essential for diabetes prevention. The American Diabetes Association (ADA) states that medical nutrition therapy (MNT) is important in the overall diabetes treatment strategy and the need for MNT should be re-evaluated periodically by health professionals in conjunction with individuals with diabetes during their lifetime (37). Further, improved awareness of diabetes following prompt detection is critical for diabetes management (5). The awareness of nutritional values labeled on food items may improve dietary intake habits (38).

Among 1,433 subjects with diabetes, 130 received nutrition education (9.1%), without any significant difference between groups. The total energy and cholesterol intake appeared to be higher in the group without nutrition education. Thus, the lack of nutrition counseling is a major concern among participants in the diabetes-aware group. In a previous study, the analysis of 1,904 diabetic subjects who participated in the 2008–13 KNHANES study in Korea (uncontrolled and well-controlled group) revealed that 15.9% individuals acquired diabetes education and 14.4% received nutrition education in the well-controlled group. The study found a positive association between nutrition education and glucose control (19). A recent study from Iran showed that nutrition education of older people with type 2 diabetes improves dietary behaviors and awareness (39). These results underscore the need for strengthening nutrition education in the general and diabetic population of Korea.

Among diabetes-aware individuals, retinol (*P* = 0.0137) intake was higher in the treatment group compared with control, although a low intake was found in the nutrition education group relative to individuals who were not exposed to nutrition education. A recent review of human studies reported an inconsistent association between retinol and diabetes (40). Another study reported that a higher dietary intake of retinol was inversely correlated with the risk of diabetic nephropathy (41). Thus, further direct studies evaluating the effect of retinol and its equivalent on diabetes are required. The intake of omega-3 fatty acid was greater in the treatment group (*P* = 0.0211) than in those exposed to nutrition education where intake was lower than in the uneducated group. The higher omega-3 fatty acid intake may be attributed to its important role in diabetes prevention and treatment. However, existing evidence based on 83 randomized controlled trials suggested little-to-no impact of increasing omega-3 and−6 fatty acids, or complete polyunsaturated fatty acids on type 2 diabetes prevention and treatment (42).

Individuals in diabetes aware group had lower intakes of riboflavin (*P* = 0.0035) and niacin (*P* = 0.0228) compared with unaware controls. Similar results were found in subjects exposed to nutrition education and treatment compared with uneducated and untreated controls, although no significant difference was observed. These findings are consistent with a previous study (28), where individuals in the aware group reported lower intakes of these vitamins, without any statistical difference. A recent study based on the National Health and Nutrition Examination Survey (2007–14) suggested that higher intakes of vitamin B1, riboflavin, niacin, B6 and dietary folate equivalent were associated with reduced risk of metabolic syndrome (43).

According to the WHO, the global maximum intake limit for sodium is 2,000 mg/day in adults (44). In this study, the sodium intake was found to be lower in the diabetes-aware group, nutrition education group, and treatment group compared with unaware, uneducated and untreated controls without any significant difference between groups. The findings are consistent with a previous study (28), where the aware group had a lower intake of sodium compared to unaware control without any significant difference. Higher sodium intakes were shown to be significantly correlated with levels of fasting glucose and HbA1c in a dose-dependent fashion leading to a higher incidence of diabetes in a prospective analysis by Hao et al. (45). In the present study, despite low sugar intake, a significantly higher proportion of HbA1c was observed in the treatment group than in control (Figure 2), underscoring the need for implementation of nutrition education, especially for diabetic patients undergoing treatment. The non-drug treatment group showed a slightly higher exercise practice rate, but the smoking and drinking rates were higher, suggesting the role of other factors. Individuals without drug treatment earn a lower average income than individuals with drug treatment, which suggests the importance of diabetes awareness and nutrition education among lower socioeconomic classes. Based on 2013–16 KNHANES data, the rates of HbA1c < 6.5% (39 mmol/mol), HbA1c < 7.0% (52 mmol/mol), and HbA1c ≥ 8.0% (64 mmol/mol) were 25.1, 52.6% and 20.9%, respectively, in diabetic individuals (5).

It has been observed that two-thirds of the diabetic patients monitored at primary care clinics in Korea undergo periodic HbA1c testing and most of the patients do not meet the existing clinical practice recommendations endorsed by the KDA (46). A new study involving diabetes prevention stipulated educational programs including, nutrition counseling, which were associated with a lower risk of diabetes, based on FBG, weight, HbA1c, and 2-h blood glucose levels. A meta-analysis revealed that the dietitians played a better role in diabetes prevention than non-dietitian providers (47).

Hence, appropriate diabetes management can reduce diabetes-related risks and mortality. Nutrition awareness among patients and the general population to improve instructional programs for training and planning of medical professionals cannot be overstated. Patients with diabetes and their kith and kin should learn and practice healthy lifestyle, including monitoring of blood sugar, following medication instructions, ensuring proper diet, and regular physical activity. These behaviors are important both in controlling diabetes and in preventing or delaying its complications.

The strength of this study is to use the most recent nationwide community-based data in Korea to determine the level of diabetes, awareness of diabetes, nutrition education, and treatment or not in Korean adults. Despite these strengths, study limitations include a one-day food intake assessment that was used as a nutrition survey. So, there is a possibility that nutrient intake cannot be reflected as the usual intake of nutrients. Second, the findings of the present study may not be generalizable to people of various ethnic groups and races because genetic makeup and lifestyle factors may influence the outcomes.

## Data availability statement

The data used in our study has been archived in a public depository, it is also publicly available and ethical clearance is not needed. The IRB number for the released data from the KNHANES is 2018-01-03-P-A.

## Ethics statement

Ethical review and approval was not required for the study on human participants in accordance with the local legislation and institutional requirements. Written informed consent for participation was not required for this study in accordance with the national legislation and the institutional requirements. No human studies are presented in the manuscript.

## Author contributions

H-JL contributed to the design and conception of the study, guarantor of this work, and had direct access to all the research results and takes responsibility for the quality of the data and the accuracy of data analysis. AS analyzed the data and wrote the manuscript. H-JL, AS, and K-HS contributed significantly to the pre-submission manuscript revision. H-JL and CL contributed to data procuring and analysis. All authors contributed to the article and approved the submitted version.

## Funding

This work was carried out with the support of Cooperative Research Program for Agriculture Science and Technology Development (Project No. PJ014536022022) Rural Development Administration, Republic of Korea.

## Conflict of interest

The authors declare that the research was conducted in the absence of any commercial or financial relationships that could be construed as a potential conflict of interest.

## Publisher's note

All claims expressed in this article are solely those of the authors and do not necessarily represent those of their affiliated organizations, or those of the publisher, the editors and the reviewers. Any product that may be evaluated in this article, or claim that may be made by its manufacturer, is not guaranteed or endorsed by the publisher.
